# Biosafety, biosecurity, and bioethics

**DOI:** 10.1007/s40592-024-00204-3

**Published:** 2024-07-30

**Authors:** David B. Resnik

**Affiliations:** National Institute of Environmental Health Sciences, National Institutes of Health, 111 Alexander Drive, Research Triangle Park, NC 27709 USA

**Keywords:** Biosafety, Biosecurity, Bioethics, Risk, Democracy, Gain of function research

## Abstract

The COVID-19 pandemic has highlighted the importance of biosafety in the biomedical sciences. While it is often assumed that biosafety is a purely technical matter that has little to do with philosophy or the humanities, biosafety raises important ethical issues that have not been adequately examined in the scientific or bioethics literature. This article reviews some pivotal events in the history of biosafety and biosecurity and explores three different biosafety topics that generate significant ethical concerns, i.e., risk assessment, risk management, and risk distribution. The article also discusses the role of democratic governance in the oversight of biosafety and offers some suggestions for incorporating bioethics into biosafety practice, education, and policy.

## Introduction

Over seven million people have died from COVID-19 in the last four years, despite the concerted efforts of governments, public health officials, medical professionals, and biomedical researchers to slow down the spread of the disease and save lives (World Health Organization 2024). While science, medicine, and technology have been instrumental in controlling this pandemic, they have been less effective than one would have hoped. Although COVID-19 has not been as devasting as the Spanish flu of 1918, which killed an estimated 50 million people, it has demonstrated, in sobering terms, that the human population in the twenty-first century is still vulnerable to highly transmissible infectious diseases (Centers for Disease Control and Prevention 2018; Young 2023).

In thinking about how to prevent the next pandemic, researchers and policymakers have discussed various problems that hampered efforts to control COVID-19, including inadequate testing, surveillance, and case reporting; lack of public trust in science and medicine; poor adherence to safety guidelines; politization of public health; conflicts between policies at the local, national, and international level; insufficient production of protective equipment and medical supplies; and difficulties with equitable and effective distribution of vaccines and treatments (Stolberg and Weiland 2023; Marcassoli et al. 2023).

A topic that has probably not received enough attention is lack of vigilance concerning biosafety (Young 2023). Concerns about biosafety entered the public forum because of the scientific debate about the origins of the pandemic. While the majority of scientists initially believed that the virus that causes COVID-19, SARS-CoV-2, evolved in an animal population in China (possibly horseshoe bats) and entered the human population by means of one or more intermediate host species (possibly animals being sold at the Wuhan wet market), this hypothesis has not been conclusively proven and many scientists now believe that the virus may have escaped from the Wuhan Institute of Virology (WIV) in the fall of 2019 (Andersen et al. 2020; Haider et al. 2020; Sirotkin and Sirotkin 2020; Wade 2021; Ridley and Chan 2021; Senate Committee on Health Education, Labor, and Pension 2022; Mallapatay 2023; Lewis 2023; Director of National Intelligence 2023; Calvert and Arbuthnutt 2023; Chan 2024).

Although most proponents of the lab escape hypothesis claim that SARS-CoV-2 was the product of genetic engineering or serial passaging^1^ experiments to enhance the virulence of a naturally occurring coronavirus, one does not need to make this assumption to think that SARS-CoV-2 could have entered the human population by means of a biosafety incident. It is possible that the virus was collected from animals and then accidentally infected workers who were studying it in the laboratory but not genetically enhancing it. Scientists from the WIV have been collecting, studying, and experimenting with coronaviruses since the SARS epidemic of 2003–2004, and history teaches us that coronaviruses have escaped from laboratories in the past (Kimman et al. 2008; Young 2023). According to the World Health Organization (WHO), during the SARS epidemic of 2003–2004, in which there were over 8,000 reported cases and 774 deaths worldwide, the virus escaped from a laboratory in Beijing twice, infecting nine people and killing one (Parry 2004; Walgate 2004; American Lung Association 2023). Several years prior to the COVID-19 pandemic, scientists and intelligence officials had raised concerns about biosafety problems in China’s rapidly expanding network of microbiology laboratories (Cyranoski 2017; Yuan et al. 2020; Young 2023).

We may never fully understand the origins of COVID-19 because the Chinese government has destroyed and suppressed coronavirus data and prevented WHO officials who were investigating the origins of COVID-19 from having unrestricted access to the WIV and researchers working there (Harrison and Sachs 2022; Cohen 2023; Calvert and Arbuthnutt 2023; Young 2023). Nevertheless, the idea that a biosafety lapse at the WIV—or some other laboratory for that matter—could have caused the COVID-19 pandemic is a very real possibility that has significant bioethical and public policy implications.

Most of the articles or book chapters on bio-risks written by bioethicists have focused on “hot” topics related to biosecurity, such as bioterrorism and dual use research, rather than on biosafety per se (see, for example, Kuhlau et al. 2011; Rappert and Selgelid 2013; Kaebnick et al. 2016; Holm 2017; Evans et al. 2022).^2^ While it is often assumed that biosafety is a purely technical matter that has little to do with philosophy or the humanities, biosafety raises important ethical issues that have not been adequately examined in the scientific or bioethics literature (Kambouris 2023; Johnson 2024). To provide some useful background to these ethical issues, this article will first review some pivotal events in the history of biosafety and biosecurity. The article will then explore three different biosafety topics that generate significant ethical concerns, i.e., risk assessment, risk management, and risk distribution. Finally, the article will also discuss the role of democratic decision-making in the oversight of biosafety and offer some suggestions for incorporating bioethics into biosafety. Note: the findings and recommendations in this article are based on my review of the biosafety literature and my analysis of the issues rather than on direct observations of research at the NIH.

## Defining biosafety and biosecurity

Biosafety can be defined as “the use of specific practices, training, safety equipment, and specially designed buildings to protect the worker, community, and environment from an accidental exposure or unintentional release of infectious agents and toxins (Department of Health and Human Services 2017a).”^3^ Biosafety is closely related to another concept, biosecurity, which can be defined as “the protection, control of, and accountability for high-consequence biological agents and toxins, and critical relevant biological materials and information within laboratories to prevent unauthorized possession, loss, theft, misuse, diversion, and intentional release (Department of Health and Human Services 2015).” Biosafety and biosecurity both deal with the management of biological risks; however, biosafety focuses on preventing accidental exposure to or release of dangerous biological agents and toxins, while biosecurity focuses on deliberate misuses of dangerous biological agents and toxins (Beeckman and Rüdelsheim 2020). Although this article will focus on the ethics of biosafety, it will also consider biosecurity matters, which are often closely connected to biosafety.

## A brief history of biosafety and biosecurity

Biological research has involved significant safety issues since Louis Pasteur (1822–1895), Edward Koch (1843–1910), and other pioneering microbiologists began collecting, isolating, and culturing microorganisms in the 1800s (see Table 1). However, governments did not take a strong interest in biosafety until the 1950s, when the risks of bioweapons research became apparent. During this time, both the US and the Soviet Union were conducting research on offensive bioweapons and defensive countermeasures (Frischknecht 2003). The first unofficial meeting of the American Biosafety Association (ABSA) took place at a military facility in Fort Detrick, MD in 1955. Attendees were military personnel, and the sessions were classified. In 1957, the ABSA expanded its constituency and began holding non-classified sessions, and by the mid-1960s, scientists and administrators from the Centers for Disease Control (CDC), National Institutes of Health (NIH), and other federal agencies were attending its meetings. In 1966, meeting attendees included representatives from universities, hospitals, and private industry (Connell 2011). By the early 1970s, the Occupational Safety and Health Administration (OSHA) had developed biosafety regulations (Occupational Safety and Health Administration 2011).

**Table 1:** Some key events in the history of biosafety and biosecurity

1955	First meeting of the American Biosafety Association
1972	109 nations sign the Biological Weapons and Toxins Convention
1972	Paul Berg, Stanley Cohen, and Herbert Boyer develop techniques for recombining bacterial DNA
1973–1975	Asilomar Conferences on Recombinant DNA Experiments
1974	NIH forms the Recombinant DNA Advisory Committee (RAC)
1976	The RAC Publishes Biosafety Guidelines
1979	NIH and CDC establish a guidance framework for biosafety laboratories
1979	66 people die from anthrax poisoning in an accident at a laboratory in Sverdlovsk in the former Soviet Union
1984	751 people develop salmonella poisoning from bioterrorist attacks in restaurants at the Dalles, Oregon
1986	CDC publishes the first edition of *Biosafety in Microbiological and Biomedical Laboratories*
1996	GM crops and foods become commercially available
1998–2007	The European Union bans GM crops
2000	103 nations adopt the Cartagena Protocol on Biosafety
2001	Anthrax terrorist attacks in the US kill five people and sicken dozens
2002	The US passes laws for control of select biological agents and toxins
2003–2004	One person dies and nine get sick as result of SARS escaping from a lab in Beijing
2004	The National Research Council issues an influential report, *Biotechnology in the Age of Terrorism*
2004	The US establish the National Advisory Board for Biosafety and Biosecurity (NSABB)
2011–2012	At the request of *Science* and *Nature*, the NSABB reviews two papers reporting the results of gain of function experiments involving the H5N1 avian influenza virus; the NSABB recommends publication of both papers without redaction
2014	The NIH announces a pause of funding of gain of function experiments involving potential pandemic pathogens
2014	The NIH and CDC report serious biosafety lapses
2015	Ralph Baric’s laboratory publishes a paper reporting the results of experiments involving the use of a mouse-adapted SARS-CoV backbone to create a chimeric virus that expresses the spike protein of SHC014
2017	The NIH lifts the funding pause on gain of function research and establishes guidelines for research involving enhancement of potential pandemic pathogens
2020–2023	During the COVID-19 pandemic, in which over 7 million people die, a scientific and political controversy emerges over the origins of the virus, gain of function research, and biosafety
2024	The US government strengthens its regulatory oversight of dual research, including research involving gain of function experiments on potential pandemic pathogens

In 1972, the US officially abandoned offensive bioweapons research when it signed the Biological Weapons and Toxins Convention (BWTC). Although the Soviet Union also signed the BWTC, it continued to conduct secret research on offensive bioweapons (Leitenberg and Zilinskas 1989). Very little is publicly known about what the Soviet Union did to promote biosafety during the Cold War era (Leitenberg and Zilinskas 1989). However, there are reasons to believe that its safety measures were inadequate. For example, in 1979, 66 people died from anthrax poisoning due to an accident at a laboratory in Sverdlovsk (Zhou et al. 2019), but this incident was not verified until 15 years after it happened because Soviet authorities covered it up (Leitenberg and Zilinskas 1989).

Biosafety became a major concern for molecular biologists in the early 1970s, when Paul Berg (1926–2023), Stanley Cohen (1922–2020), and Herbert Boyer developed techniques for recombining DNA from different bacteria (Jackson et al. 1972; Connell 2011). Scientists working with recombinant DNA soon realized that their experiments posed serious risks to public health and the environment if genetically modified organisms (GMOs) were to escape from the laboratory and infect people, animals, or plants. Leaders of this field met in Asilomar, CA in 1973, 1974, and 1975 to discuss biosafety issues and develop biosafety protocols. In 1974, Berg, Cohen, Boyer and other top scientists called for a voluntary moratorium on recombinant DNA experiments until these risks were better understood and appropriate safety protocols were in place (Berg et al. 1974). Berg et al. (1975) published a paper describing some biosafety principles and recommendations for recombinant DNA experiments.

In 1974, the NIH formed the recombinant DNA advisory committee (RAC) to provide guidance for NIH-funded recombinant DNA experiments. In 1976, the RAC published guidelines for recombinant DNA research, which included the requirement that funded institutions establish institutional biosafety committees (IBCs) to oversee recombinant DNA experiments. Although not required by NIH policy, most IBCs also oversee other types of dangerous biological research, such as research involving the collection and storage of deadly pathogens (Resnik 2021). Under NIH policy, IBCs must include at least five members with appropriate expertise and experience, at least two of whom must be non-institutional members who represent the interests of the local community (National Institutes of Health 2019). Institutional Review Boards (IRBs), which oversee human subjects research, and Animal Care and Use Committees, which oversee animal experimentation are also required to include non-institutional members (Shamoo and Resnik 2022). Also in 1974, the US Postal Service and the Department of Transportation promulgated regulations for shipping bacteria and other etiologic agents (Connell 2011).

By 1979, the CDC and NIH had established a guidance framework for biosafety laboratories (BSL) (Connell 2011; Beeckman and Rüdelsheim 2020). The framework includes four safety levels corresponding to the degree of risk from infectious agents or toxins. For example, the safety measures used in a BSL-1 lab, such as storing agents in sealed containers and washing surfaces, are adequate for research on benign organisms like *Escherichia coli*, but not for dangerous pathogens that pose a high risk of aerosol transmitted infection, such as the Ebola virus. To study the Ebola virus, one should use the safety measures found in a BSL-4 lab, such as wearing a full-body, air-supplied suit, working in a physically isolated laboratory, and undergoing decontamination procedures when leaving the laboratory. See Table 2. The BSL framework is used by institutions around the world to guide biosafety practices (World Health Organization 2020). In 1986, the CDC published the 1st edition of *Biosafety in Microbiological and Biomedical Laboratories* (now in its 6th edition), which describes the BSL framework in detail (Centers for Disease Control and Prevention 2020).

**Table 2:** Biosafety levels (based on Centers for Disease Control and Prevention 2020)

	Description of agents	Example	Safety requirements (sample)
Biosafety Level 1	Infectious agents or toxins not known to consistently cause disease in healthy adults	*Escherichia coli*	Standard microbiological safety practices; surfaces must be easily cleaned and can withstand basic chemicals
Biosafety Level 2	Moderate risk infectious agents or toxins that pose a risk infection if accidentally inhaled, swallowed, or exposed to skin	Influenza virus, HIV, Lyme disease	Hand washing sinks; eye washing stations; access to equipment that can decontaminate laboratory waste, including an incinerator, an autoclave, and/or another method
Biosafety Level 3	Infectious agents or toxins that may be transmitted through the air and cause potentially lethal infection through inhalation exposure	Tuberculosis	Experiments performed in biosafety cabinets; laboratories are designed to be easily decontaminated and must use controlled air flow; sealed windows and wall surfaces, and filtered ventilation systems;
Biosafety Level 4	Infectious agents or toxins that pose a high risk of aerosol-transmitted laboratory infections and life-threatening disease for which no vaccine or therapy is available	Ebola virus	Laboratories located in safe, isolated zones within a larger building or are housed in a separate, dedicated building; work may be done in a biosafety cabinet or laboratory personnel wear full-body, air-supplied suits and go through a series of procedures designed to fully decontaminate them before leaving the building

Although the public became aware of the biosafety issues related to genetic manipulation through magazine and newspaper editorials warning about the dangers of “superbugs” and popular books that became movies, such as *The Andromeda Strain* (Crichton 1969), biosafety was, at that time, more of a scientific issue than a political one. The President's Commission for the Study of Ethical Problems in Medicine and Biomedical and Behavioral Research (1982) did publish a report titled *Splicing Life*, but the document focused, for the most part, on the ethics of genetically engineering human beings and did not examine biosafety issues in much depth. The US Supreme Court’s decision in 1980 to uphold Ananda Chakrabarty’s patent on genetically modified (GM) bacteria that metabolizes crude oil met with little fanfare or public protest, even though it proved to be instrumental in stimulating the burgeoning biotechnology industry (Resnik 2004).

During the 1980s and 1990s, important steps were taken to internationalize biosafety. In 1984, the ABSA became ABSA International and began affiliating with biosafety associations around the globe (American Biosafety Association International 2023). In 2001, the International Biosafety Working Group was formed, which later became the International Federation of Biosafety Associations (2023) (IFBA). The IFBA also includes over 30 biosafety safety associations from North and South America, Europe, Asia, and Africa. An important biosafety treaty, the Cartagena Protocol on Biosafety to the Convention on Biological Diversity, was negotiated in the 1990s and adopted by 103 countries in 2000. The protocol, which has been signed by 173 countries, governs the safe transfer, handling, and use of GMOs (Cartagena Protocol on Biosafety 2023).

In 1996, the first GM crops and foods became commercially available. Soon afterward, European citizens and farmers protested against GM crops and “Frankenfoods” and the European Union banned GM foods and crops until 2008 (Resnik  2021). Although GM crops remain a controversial issue in biotechnology and over two dozen countries currently ban or significantly restrict their cultivation, most of the public concern about GM has focused on the health risks of consuming GM foods and to a lesser extent the bio-risks of growing GM crops, such as the environmental impacts of accidental or intentional releases of GM plants (Resnik 2021).

At the beginning of the twenty-first century, bioterrorism and biosecurity emerged as major concerns for science policy. In the fall of 2001, Bruce Ivins, a biodefense researcher at the US Army Medical Research Institute of Infectious Diseases in Fort Detrick, allegedly sent letters laced with anthrax spores to Senators Tom Daschle and Patrick Leahy and several members of the media. The attacks, which killed five people, sickened 17, and exposed dozens of postal workers to anthrax spores, created tremendous anxiety in a nation already reeling from the terrorist attacks of September 11, 2001. The Federal Bureau of Investigation’s lengthy and costly investigation eventually named Ivins as the suspect in 2008, but Ivins committed suicide before he could be apprehended and brought to trial (CNN 2023). It is worth noting that this was not the first bioterrorist attack on US soil. In 1984, members of a religious cult led by Bhagwan Rajneesh sprayed salmonella bacteria on salad bars in ten restaurants in The Dalles, Oregon in an effort to make people too sick to vote in Wasco County elections so that the cult’s candidates would win. 751 people developed salmonella poisoning, but fortunately no one died (Author 2021).

In response to the anthrax attacks as well as other concerns about terrorism and biosecurity, the US Congress in 2002 passed the Agricultural Bioterrorism Protection Act and the Public Health Security and Bioterrorism Preparedness and Response Act, which establish requirements for the possession, control, use, and transfer of dangerous biological entities known as select agents and toxins (Connell 2011). Currently, there are 68 types of biological entities classified as select agents or toxins, including SARS, genetically manipulated forms of SARS, anthrax, Ebola, smallpox, and reconstructed pandemic influenza 1918 (Federal Select Agent Program 2022).

During the early 2000s, several papers were published in biomedical journals that raised issues of “dual use;” that is, the results of the research could be used for beneficial purposes, such as advancing science, medicine, or public health, but also could be used for harmful purposes, such as terrorism, crime, or warfare (Miller and Selgelid 2007). Some of these publications included a study showing how to increase virulence of a virus used to vaccinate people against smallpox, a study showing how to manufacture a polio virus from DNA sequence data and mail-order supplies, a study describing a mathematical model for infecting the US milk supply with botulinum toxin, and study showing how to reconstruct the extinct 1918 Spanish flu virus (Rosengard et al. 2002; Cello et al. 2002; Wein and Liu 2005; Tumpey et al. 2005).

These dual use issues caught the attention of the US Congress, which asked the National Research Council (NRC) to study them. In 2004, the NRC issued an influential report, *Biotechnology in the Age of Terrorism*, which examined the ethical and policy issues raised by these and other publications and identified some types of research that warrant special scrutiny, including experiments that attempt to enhance the harmful consequences of a biological agent or toxin, disrupt the effectiveness of an immunization against a biological agent, increase the transmissibility of a biological agent or toxin, or create a novel biological agent or toxin or reconstitute an eradicated biological agent or toxin (National Research Council 2004; Shinomiya et al. 2022). The NRC also recommended that the US government form an organization to provide scientists, journal editors, and government officials with guidance and advice on biosecurity and biosafety issues.

The US government followed the NRC’s recommendation and formed the National Science Advisory Board for Biosecurity (NSABB) in 2004 (National Science Advisory Board for Biosecurity 2023). Because “dual use research” is potentially very broad in scope, the NSABB changed the term to “dual use research of concern” (DURC) to focus the scope of ethical and policy debate (World Health Organization 2010). The US government currently defines DURC as “[L]ife sciences research that, based on current understanding, can be reasonably anticipated to provide knowledge, information, products, or technologies that could be misapplied to do harm with no, or only minor, modification to pose a significant threat with potential consequences to public health and safety, agricultural crops and other plants, animals, the environment, materiel, or national security (The Whitehouse 2024).”

The most controversial case the NSABB has examined thus far involved two NIH-funded studies describing how to genetically modify the H5N1 avian influenza virus to enable it to be transmissible between mammals by respiratory water droplets. The papers describing the studies were submitted to *Science* and *Nature* in 2011 (Russell et al. 2012; Imai et al. 2012). One of the studies was headed by Ronald Fouchier at Erasmus Medical Center in the Netherlands and the other by Yoshihiro Kowoaka at the University of Wisconsin-Madison. The journals editors gave the papers special review because they recognized that the results of the research could be used to make a bioweapon and asked the NSABB to render an opinion as to whether these papers should be published. At its December 2011 meeting, the NSABB recommended, by unanimous vote, that neither paper should be published in full. The papers could be published only if key information needed to replicate the experiments was redacted (Resnik 2013). The primary basis for the NSABB’s opinion was that the biosecurity risks of publication could not be justified on the basis of the benefits of the research for science and public health, such as potential applications in the development of treatments or vaccines or monitoring of bird populations for dangerous mutations. The NSABB was also concerned about the biosafety risks of publication if others tried to replicate the experiments, but these apprehensions were secondary, at least initially.

After the NSABB’s ruling, researchers working in this area agreed to a temporary moratorium on experiments that enhance the virulence or transmissibility of avian influenza viruses. In February 2012, the WHO convened a workshop to examine the issues raised by the papers and recommended full publication. In March 2022, after reading longer versions of the papers that included additional information about biosecurity and biosafety protocols and the benefits of publication, the NSABB reversed its earlier ruling and recommended full publication of both papers. However, a substantial minority of NSABB members felt that the research conducted Fouchier’s laboratory (Russell et al. 2012) should be published only in redacted form (Author 2013).

Later in 2012, after the publication of the controversial papers, the US government adopted a policy for the oversight of DURC in the life sciences research funded by federal agencies. The policy defined DURC and set forth procedures agencies must follow in assessing and mitigating the risks of DURC. The policy only applied to seven categories of dangerous experiments involving biological entities on the selects or toxins list (United States Government 2012).

The announcement of a new US government DURC policy did not quell the debate about genetic manipulation of dangerous pathogens. Many scientists and some bioethicists argued that the benefits of this research were not worth the risks and urged researchers to consider alternative approaches to studying the genetic-molecular structure, evolutionary dynamics, and pathogenicity of viruses (Wain-Hobson 2013; Lipsitch and Inglesby 2014; Evans et al. 2015). Other scientists pushed back against these critiques by arguing that the research has important benefits for medicine, public health, and science and that the risks can be managed with appropriate safeguards and security (Fauci 2012; Casadevall et al. 2014; Davis et al. 2014). The NSABB and NRC held several meetings to consider these issues in more depth and solicit input from scientists and members of the public.

An important development that occurred at this time is that the biosafety risks of research involving dangerous pathogens began to be seen by many as just as important as biosecurity risks, due in part, to concerns raised by scientists at NSABB and NRC meetings and some biosafety lapses in federal laboratories (Shinomiya et al. 2022). For example, in July 2014 six vials of variola virus, which causes smallpox, were discovered during an inventory of materials stored in a Food and Drug Administration (FDA) cold storage room at the NIH (Young 2023). In December 2014, the CDC reported that some material from an Ebola experiment was improperly transported from a BSL-4 lab to a BSL-2 lab (Centers for Disease Control and Prevention 2015). Although these incidents did not expose laboratory workers to deadly pathogens, the fact that these materials were not properly stored, transferred, or accounted for was a major biosafety problem. The NIH and CDC took measures to improve biosafety practices and oversight as a result of these incidents (Young 2023).

In October 2014, DHHS announced a pause on new funding of gain of function (GOF) genetic manipulation experiments involving SARS, MERS, and other potential pandemic pathogens (PPPs) and asked the NSABB to review the issues and make policy recommendations. GOF research is research that attempts to genetically manipulate a pathogen to enable it to acquire a new function, such as increased transmissibility or virulence (NRC 2015). The NSABB recommended that the DHHS should lift the funding pause after implementing an oversight framework that minimizes and manages the risks of GOF research with PPPs. The DHHS followed these recommendations and lifted the funding pause on December 2017. The DHHS’s oversight framework established criteria for making funding decisions for research involving genetic enhancement of PPPs and outlines responsibilities for funding agencies and DHHS. The framework defined a PPP as a pathogen that “is likely highly transmissible and likely capable of wide and uncontrollable spread in human populations; and…is likely highly virulent and likely to cause significant morbidity and/or mortality in humans (Department of Health and Human Services 2017b).” The framework defined an enhanced PPP as “a PPP resulting from the enhancement of the transmissibility and/or virulence of a pathogen. Enhanced PPPs do not include naturally occurring pathogens that are circulating in or have been recovered from nature, regardless of their pandemic potential (Department of Health and Human Services 2017b).”

In 2015, an NIH-funded research team led by Ralph Baric at the University of North Carolina at Chapel Hill published a paper reporting the results of an experiment in which they used a mouse-adapted SARS-CoV backbone to create a chimeric virus that expresses the spike protein of SHC014. The virus was able to use angiotensin-converting enzyme 2 (ACE2) receptors to infect and replicate in human airway cells and caused pathogenesis in humanized mice. The authors hypothesized that coronavirus pools found in horseshoe bats maintain spike proteins that give them the capability of infecting humans and stated that there is “a potential risk of SARS-CoV re-emergence from viruses currently circulating in bat populations (Menachery et al. 2015: 1508).” WIV researcher Shi Zhengli, the second to last author on the paper who had been collecting bat coronaviruses since 2004, provided the genomic sequence for the spike protein. Shi has conducted similar experiments at the WIV. While Baric’s research was conducted in a BSL-3 lab, Shi’s was conducted in a BSL-2 lab (Jacobsen 2021).

Although the study did not generate much public interest when it was published, it became controversial in May 2021, when US Senator Rand Paul questioned NIAID Director Anthony Fauci about the study during Senate Health Committee hearings. Senator Paul claimed that the study was a GOF experiment that violated the NIH’s funding pause, but Fauci denied this allegation. Paul also said that the NIH had funded Shi’s research at the WIV through grants to EcoHealth Alliance, but Fauci also denied this claim. However, the NIH later admitted that it had funded Shi’s research but was unaware that this had happened because of EcoHealth Alliance’s lack of transparency and accountability concerning its research projects (Basu 2021; Shinomiya et al. 2022; Calvert and Arbuthnutt 2023). In May 2024, the NIH terminated EcoHealth Alliance’s funding due to its lax oversight and reporting of research (Lenharo 2024).

Since 2014, public health and governmental organizations have held meetings to discuss ways of enhancing biosafety (Shinomiya et al. 2022). The ABSA’s annual meetings have brought together hundreds of scientists, public health practitioners, and government officials from all over the world (ABSA 2023). In December 2017, the WHO (World Health Organization 2017) held a meeting that included experts from more than 20 countries and 53 institutions to discuss challenges, opportunities, and solutions for maximizing safety in BSL-4 laboratories. In October 2020, the US government hosted a meeting attended by scientists and administrators (but no ethicists) from various federal agencies including DHHS, NIH, FDA, USDA, DOE, FBI, and EPA, to discuss policies and strategies for enhancing biosafety. The meeting led to the publication of a report in 2022 (National Science and Technology Council 2022).

At the beginning of May 2024, the Biden Administration announced that it had revised federal policies DURC and PPP research (The Whitehouse 2024; Kaiser 2024). The new policies were adopted in response to pressures from politicians and members of the public who were concerned about the risks of GOF and the possibility that the pathogen that caused the COVID-19 pandemic was a genetically manipulated virus that escaped from the WIV. The policy expanded the types of research that require review and oversight and revised the definition of a PPP to include a pathogen that could have pandemic potential as result of being modified. The policy distinguishes between two categories of research: 1) DURC research, including nine types of experiments of concern, similar to those identified by the NRC; and 2) research involving PPPs or PPEPs (potential pandemic enhanced pathogens). A PPP is defined as a “pathogen that is likely capable of wide and uncontrollable spread in a human population and would likely cause moderate to severe disease and/or mortality in humans (The Whitehouse 2024). The policy also provides guidance for policy implementation.

## Bioethics issues

From this brief history, we can see that biosafety and biosecurity have been an important topic in science policy since the 1950s. However, although discussions of biosecurity in the professional literature and public meetings have addressed ethical issues, discussions of biosafety have focused mostly on scientific and technical issues. With the notable exception of dual use research, there has been little interaction between bioethicists and bio-scientists on matters of biosafety. As noted earlier, an important biosafety meeting held by the US government in 2020 did not include any ethicists. While the NSABB has included ethicist members and has featured presentations by ethicists, most of the discussion at these meetings has centered on scientific and technical issues (National Science Advisory Board for Biosecurity 2005, 2014, 2023). Although there is little doubt that scientific and technical issues should take centerstage in discussions of biosafety, ethical issues also deserve serious attention, because safety has implications for human wellbeing, human rights, justice, and other moral values. Some important ethical issues in biosafety are described below (see Table 3).

**Table 3:** Ethical issues related to biosafety

Policy domain	Issues
Risk assessment	What are the risks and benefits of the research?
	Are the risks of the research acceptable, given the potential benefits?
	How should decision-makers deal with uncertainty concerning risks and benefits?
Risk management	How should the risks of the research be managed?
	Should some studies not be published in full?
	Who should have access to dangerous materials?
	What BSL level is appropriate for an experiment?
Risk distribution	How are the risks and benefits of the research expected to be distributed?
	Who will face the most significant risks?
	Who stands to benefit?
	Is the expected distribution fair?
	Will the local communities and the public have appropriate input into decisions concerning the acceptability, management, and the distribution of risks?

### Risk assessment

Risk assessment is the most fundamental ethical issue related to biosafety and biosecurity because research should not be conducted and funded^4^ if it is likely to create risks that are unacceptable. Risk assessment consists of three parts: (1) identifying potential risks and benefits of a human activity, technology, or other item to be assessed; (2) assigning probability estimates to risks and benefits; and (3) weighing risks and benefits. Risk assessment requires the exercise of ethical (or moral)^5^ judgment because the third part involves deciding whether risks are ethically justifiable, given the benefits (Resnik 2021). Risk assessment is a fundamental safety issue in bioscience, regardless of whether the research involves genetic experiments that create novel agents or toxins or only entails the collection, observation, and storage of naturally occurring pathogens, such as SARS or Ebola.

While there has been some discussion in the bioethics literature about how to assess the risks and benefits of research involving genetic manipulation of pathogens (see Kuhlau et al. 2011; Resnik 2013; Selgelid 2016), there has been very little discussion about assessing the risks of biological research that does not involve genetic manipulation, such as the research that led to the SARS accident in a lab in Beijing in 2004, which presumably did not involve genetic manipulation.^6^ For several decades, scientists have considered arguments for and against destroying stockpiles of smallpox, but this debate has taken place mostly without input from bioethicists (Weinstein 2011; Gronvall and Sell 2022). Although Selgelid (2004) has written about ethical issues related to smallpox, he has focused on public health and medical issues, such as mandatory vaccinations and informed consent, and not on the issue of whether smallpox should be stored and studied in the lab.

Since most biological research conducted in BSL-3 and BSL-4 labs does not involve the genetic manipulation of pathogens (Kimman et al. 2008), it is important for biomedical researchers, bioethicists, policymakers, and members of the public to participate in assessments and decisions related to all types of dangerous research biological research to ensure that risk/benefits issues are adequately addressed. Democratic processes (discussed in more depth below) should play an essential role in these deliberations.

One of the key principles of risk assessment is that risks should be proportional to benefits (European Commission 2000; Nuffield Council 2007; Resnik 2021). While most people would view this as a good idea, deciding whether risks are proportional to benefits is a complex determination that requires one not only to ethically compare risks and benefits but also to estimate the probabilities related to different possible outcomes. However, probability estimates concerning risks and benefits are often fraught with uncertainty, due to lack of reliable and accurate data from studies published in peer-reviewed journals or other credible sources of evidence (Resnik 2021).

How to deal with uncertainty regarding risks and benefits raises challenging ethical issues for biosafety and biosecurity (Selgelid 2016; Resnik 2021). Biosafety and biosecurity risks can be difficult to estimate due to lack of high-quality, interpretable data concerning laboratory accidents, laboratory acquired infections, security breaches, and other adverse outcomes. While there is some data concerning biosafety risks in different types of laboratories, it is difficult to interpret due to the diversity of reporting systems and metrics (Kimman et al. 2008; National Research Council 2015; Wurtz et al. 2016; Himmel 2023; Young 2023). Biosecurity risks are difficult to estimate because bioterrorism events are very rare, and predictions must therefore be made based on mathematical models, which are subject to various biases (Resnik 2021; Himmel 2023).

Because good evidence is lacking, experts often disagree significantly on the risks and benefits of dangerous biological research. For example, experts disagreed by several orders of magnitude concerning the biosafety risks of the controversial H5N1 experiments reviewed by the NSABB in 2011 (see Lipsitch and Bloom 2012; Fouchier 2015; Fouchier et al. 2012; Klotz 2015). Recently, the Department of Homeland Security (DHS) and the National Academies of Science, Engineering, and Medicine disagreed about the biosafety risks of a USDA lab built in Manhattan, Kansas (Cornwall 2023). (Further discussion below.) Experts have also disagreed about whether GOF research is likely to yield important benefits, such as knowledge that could be useful in developing treatments or vaccines (Fauci 2012; National Research Council 2015; Lipsitch 2018; DiRita and Bertuzzi 2021). When faced with uncertainties related to biological risks, some have advocated for risk-aversive policies, while others have sought to judiciously balance risks and benefits. Deciding which path to take is an ethical, not a scientific decision (Selgelid 2016; Resnik 2021).

Scientists, politicians, and members of the public also disagree about how to compare benefits and risks. Comparing risks and benefits raises moral and political questions because it involves deciding upon the level (or degree) of benefit that is required to justify serious risks, such as risks related to biosafety or biosecurity (Resnik 2021). As noted above, since 2010, some scientists (e.g., Fauci 2012) have argued that the risks of GOF are worth the benefits, while others defended the opposite viewpoint (Lipsitch 2018). Because risk estimates are fraught with uncertainty, disagreements about the justification of GOF experiments may affect these estimates, because proponents of GOF may underestimate the risks while proponents may overestimate the risks (Resnik 2021). Thus, it may be very difficult to separate “facts” from “values” when it comes to assessing biosafety and biosecurity risks.

A key assumption that supports arguments for conducting dangerous biological research is that the risks can be well-managed through implementation of appropriate biosafety and biosecurity measures (Selgelid 2016). But can they? And what ethical issues does risk management raises? These questions bring us to our next topic.

### Risk management

Risk management requires the exercise of ethical judgment because it involves deciding how to minimize or mitigate the risks of an activity once a decision has been made to engage in it, and strategies for minimizing or mitigating risks often create tradeoffs among competing values (Resnik 2021). Deciding whether to publish DURC entails tradeoffs between values, such as promoting progress in science and technology vs. preventing risks to public health, agriculture, or national security. As we saw earlier, the NSABB considered these and other values when it recommended that the H5N1 GOF studies should be published. Although the NSABB considered the option of redacted publication, it did not ultimately recommend it. However, redacted publication of DURC has occurred at least once. In 2014, a research team led by Stephen Arnon from the California Department of Public Health published a paper in which they redacted some genomic sequence data needed to make a novel *Clostridium botulinum* toxin (Dover et al. 2014). They chose to redact this information because they were concerned that someone could use it to make a bioweapon for which there was no effective treatment (Relman 2014).

Bioethicists have published articles about and taken part in NSABB sessions on publication, data sharing, and transparency issues related to DURC (see Resnik 2013, 2020; Selgelid 2016). However, important ethical questions can arise prior to publication when one is deciding a) how much safety is sufficient for conducting a study that uses or makes dangerous biological agents or toxins, and b) the type of safety measures that should be implemented in the study. These questions are conceptually similar to those that arise in other areas of risk management in science that involve competing values, such as clinical trial design (Shamoo and Resnik 2022).

As noted earlier, the BSL framework requires that the level of biosafety should correspond to degree of risk of the agent or toxin. However, deciding which BSL level to use for an experiment is not a purely technical matter because it involves tradeoffs between risks, costs, and benefits (Kimman et al. 2008). BSL-4 labs cost more money to build and operate than BSL-3 labs; BSL-3 cost more than BSL-2; and BSL-2 cost more than BSL-1. Research in BSL-4 labs is more difficult to conduct than research in BSL-3 labs because it involves the use of uncomfortable protective gear that can hinder movement (Kimman et al. 2008; Butler 2009). Additionally, some types of agents or toxins may not be easily categorized within the BSL framework, which implies that safety measures may need to be determined on a case-by-case basis (Kimman et al. 2008). Given these financial and logistical realities, researchers and funding agencies may be tempted to skimp on biosafety to save money and reduce inconvenience (Young 2023). These cost/risk/benefit issues also have implications for the distribution of risks (discussed below) because countries that cannot afford (or do not want to spend money on) BSL-3 or BSL-4 labs may create higher risks than countries that can afford to these labs if they decide to conduct risky biological research in BSL-1 or BSL-2 labs, which may have happened with coronavirus research at the WIV (Young 2023). Deciding who should have access to select agents and toxins also raises ethical issues. Access to materials raises ethical concerns because denial of access should be based on the security risks that a person poses and not on racial/ethnic, political, or other irrelevant factors. However, it can be difficult to distinguish between relevant and irrelevant factors when a making decision concerning access to research materials, especially give legitimate concerns about preventing foreign countries from stealing intellectual property, new technologies, or proprietary data (Gilbert 2023). Background checks for people who want access to select agents or toxins should be fair, judicious, and prudent.

There is widespread agreement among experts that laboratory culture plays a crucial role in promoting safe biological research (Perkins et al. 2019). The culture of a laboratory or research group can be equated with the values, beliefs, and behaviors of its members (Schneider et al. 2013). A safety culture is one in which researchers not only follow safety protocols but also believe in the importance of safety and advocate for it (Institute of Medicine 2000; Perkins et al. 2019). Questions about how to foster a safe laboratory culture are not technical/scientific issues but are humanistic ones related to education in the responsible conduct of research (RCR) (National Academies of Sciences, Engineering, and Medicine 2017; Antes et al. 2019; Shamoo and Resnik 2022). The NIH requires that RCR instruction for funded students includes education on “safe research environments,” which is equated with environments “that promote inclusion and are free of sexual, racial, ethnic, disability and other forms of discriminatory harassment (National Institutes of Health 2022).” However, one could argue that RCR education should address not only psychosocial safety but also biosafety and laboratory safety (Shamoo and Resnik 2022). Moreover, there may be situations in which safety issues intersect with other RCR issues, such as authorship, collaboration, and mentoring. For example, if a trainee and their supervisor have a disagreement about the level of safety required to conduct an experiment, this could impact the trainee’s relationship to their supervisor and their career.

The Institute of Medicine (IOM) (2000), in its groundbreaking report, *To Err Is Human: Building a Safer Health System*, emphasized the importance of fostering a safety culture in health care for preventing medical errors. The report stressed that while it is unrealistic to expect people to be error-free, the culture and system can be improved, so that mistakes can be prevented, detected, and corrected. One of the characteristics of US medical culture, according to the IOM, is that people are often reluctant to report, admit to, or share information about errors due to concerns about legal liability. The IOM recommends changing this culture so that people are more willing to report errors, admit to them, and share information about errors. To help change this culture, 29 US states have passed “I’m sorry” laws that require health care providers to disclose errors to patients and make apologies inadmissible in court as evidence of fault (Bender 2007). The report recommended improving the system by developing procedures that include multiple levels of error-prevention/detection, such as double-checking of prescriptions, blood types, known allergies, and surgical sites.

These lessons from the IOM report clearly apply to biosafety. When a biosafety accident or breach occurs, researchers and administrators must not ignore it, deny it, or cover it up but be willing to report it to the relevant authorities so that corrective actions can be taken to protect workers and the public and prevent similar events from happening in the future (Young 2023; Haines and Gronvall 2023). Although researchers are not likely to face legal liability for mistakes they make in the lab, they may still face institutional sanctions that could discourage reporting. Biological laboratories can make their cultures safety-oriented by treating self-reporting of mistakes and cooperation with an investigation as factors that mitigate or eliminate potential sanctions (Feeser et al. 2021). Additionally, biosafety protocols should include multiple levels of error-prevention/detection, such as double-checking of labels on containers, regular auditing of inventories of agents and toxins, and (if appropriate) antibody testing of workers for infections, even when they display no symptoms of disease. While it is not possible to prevent all needle sticks, spills, failures of protective gear and equipment, and other accidents in the lab, one can be prepared to respond to them when they happen (Young 2023).

Accreditation also can play a major role in promoting safety-oriented cultures and systems in biomedical research. Since 1965, the Association for Assessment and Accreditation of Laboratory Animal Care (AALAC) International has been accrediting research organizations that perform experiments on animals (Gettayacamin and Retnam 2017). Currently, over 1000 research organizations from more than 50 countries are accredited (Association for Assessment and Accreditation of Laboratory Animal Care International 2023). AAALAC International evaluates organizations based on their compliance with standards, practice, and procedures described in the National Research Council’s (2011) *Guide to the Care and Use of Laboratory Animals*. During the accreditation process, AAALAC International visits research institutions, inspects animal facilities, examines animals, evaluates the institution’s standard operating procedures for protecting animal welfare, reviews animal use protocols approved by the ACUC and ACUC meeting minutes, and interviews researchers and institutional officials. There is little doubt that AAALAC International accreditation plays ain indispensable role in promoting laboratory animal welfare (Gettayacamin and Retnam 2017).

Although there are some organizations that provide biosafety accreditation for research institutions or laboratories, there is no organization in the biosafety realm with the influence or prestige of AAALAC (National Research Council 1989; Mourya et al. 2017). The advent of such an organization could be instrumental in promoting biosafety worldwide (Silver 2022).

### Risk distribution

Another important ethical issue is the distribution of biological risks. Risk distribution raises ethical issues because one might ask whether the distribution is fair or just (Resnik 2021). Researchers working with dangerous human pathogens are likely to face the highest risks, but people living near the lab may also face risks if biological agent escapes. The sphere of risk can expand outward once an agent enters human, animal, or plant populations (Lipsitch and Bloom 2012). Although much of the recent biosafety debate has focused on risks to human health, biological agents may pose risks to non-human species, such as livestock, crops, or native plants or animals. Many pathogens, such as SARS and influenza viruses, pose risks to humans and animals. Approximately 60% of human diseases can be transmitted to animals and about 75% of new human infectious diseases come from animals (Centers for Disease Control and Prevention 2021). GM plants or animals, especially those that incorporate a gene drive system,^7^ can pose a significant threat to agriculture and ecosystems if they are accidentally or intentional released into the environment (National Academies of Sciences, Engineering, and Medicine 2016; Esvelt and Gemmell 2017).

Fairness can be understood in terms of 1) the outcomes of a distribution of risks and benefits or 2) procedures or processes used to distribute risks and benefits (Rawls 1971). For example, in asking whether a state government’s decision about where to locate a hazardous waste site was fair, one can ask whether the outcome of the decision (i.e., where the hazard was placed) was fair, or whether the procedures that the government followed in making the decision were fair (Resnik 2012). There are many different theories about what makes a distribution of risk and benefits fair or unfair, including utilitarian, egalitarian, and libertarian approaches (Barry 1991; Lamont 2017). For example, a state might select a site for hazardous waste storage with an eye toward minimizing risks for whole population or toward minimizing risks for socioeconomically disadvantaged members of the population (Resnik 2012).

While there is not sufficient space in this article to make substantive comments about what makes the outcome of a risk/benefit distribution fair or unfair, there is enough space to address the fairness of procedures. To address procedural fairness issues, it will be useful to distinguish between three groups of people: (1) people who participate in work involving dangerous biological agents or toxins; (2) people who live in the vicinity of the work; and (3) members of the greater public.

Many different activities that people engage in, including work, recreation, and travel, involve significant hazards or risks.^8^ Valid consent is a key requirement, but not the only requirement,^9^ for regarding occupational exposure to hazards and risks as fair.^10^ It is fair, one might argue, for a firefighter to encounter higher occupational risks than a librarian because the firefighter has freely and knowingly consented to these risks (Author 2012). By the same reasoning, it follows that participation in work involving significant biological hazards or risks is ethical and procedurally fair when it is consented to. To promote informed choice related to bio-risk exposure, overseers of the laboratory, such as senior investigators, must inform workers (e.g., students, postdoctoral fellows, and technicians) about biological hazards and measures being taken to reduce and control risks, such as safety protocols, protective equipment, disinfection and decontamination procedures, health monitoring, and waste disposal. Dissemination of safety information is required by OSHA regulations and is a standard practice for training researchers who work in biological laboratories (National Research Council 1989; Kimman et al 2008).

Consent may be compromised, however when people are coerced, manipulated, or deceived into working with dangerous biological agents or toxins, which could occur if a research organization outsources dangerous biological research to a low-income country or any nation, state, or region with a poor track-record of protecting human rights or worker safety. Although it is not known how often the outsourcing of dangerous biological research occurs, this is potentially an important ethical and political issue related to biosafety, especially since pharmaceutical and biotechnology companies often outsource research to minimize costs and regulatory burdens (Shah 2006; Schroeder et al. 2017).

### The role of democracy in biosafety oversight

While informed consent can promote procedural fairness for those who choose to engage in work involving biological hazards and risks, it does not apply to people living in the vicinity of the laboratory or members of the greater public, who may not have consented to taking these risks (Resnik 2014; Kolopack and Lavery 2017). How can risks to people outside the laboratory be justified? A reasonable answer to this question is that society can makes decisions concerning the acceptability and distribution of these risks through democratic processes. Democracy helps to foster procedural fairness by giving citizens equal rights to enact and shape the laws and policies that apply to them. Democracy is “consent” of the governed (for further discussion of democracy and consent, see Gutmann and Thompson 1998; Rawls 2005; Christiano and Bajaj 2022).

Political theorists distinguish between direct democracy, in which citizens vote on laws that affect them, and representative democracy, in which citizens elect representatives who make and execute laws on their behalf. Elected representatives may, in turn, appoint people to help them execute laws, and these appointees may hire other people, and so on. Because direct democracy is an impractical form of decision-making for a large group, most democratic nations and states are representative democracies. However, even representative democracies may include forms of direct democracy; for example, when citizens vote on referenda (Gutmann and Thompson 1998).

Most of today’s representative democracies include many layers of bureaucracy that intercede between the public and government actions (Bertsou and Caramani 2021). For example, the person who is charged with executing US federal environment laws, the EPA Administer, is appointed by the President and confirmed by the Senate. The EPA Administrator appoints other administrators, who hire employees and solicit expert opinion. Although the public has oversight of the EPA, most of the agency’s key decisions are made by employees, contractors, or volunteers with expertise in toxicology, epidemiology, medicine, statistics, and other relevant disciplines (Brown 2009; Resnik 2012). The public is not entirely excluded from this governance structure because expert panels often include public members and administrators usually consult with members of the public. Other agencies, such as the NIH, CDC, and FDA, implement a similar form of bureaucratic decision-making.^11^

Because powerful corporations, wealthy individuals, and political or industry groups can significantly influence government decisions by lobbying elected or appointed officials, making contributions to campaigns, or challenging laws or regulations in court, many theorists argue that representative democracies should incorporate deliberative forms of democracy to help ensure that government decisions are fair. Deliberative democracy consists of various methods for promoting in-depth discussion, thoughtful debate, and inclusive dialogue about government actions (Gutmann and Thompson 1998, 2004; Fishkin 2011). Some ways of practicing deliberative democracy include holding town hall meetings and listening sessions, sponsoring public debates, and soliciting comments on proposed laws, regulations, or decisions (Gutmann and Thompson 2004; Fishkin 2011). Community and public engagement (discussed below) are forms of deliberative democracy (Resnik 2018; Neuhaus 2018).^12^

Transparency and openness are essential to all forms of democratic governance. That is, citizens must be able to access, use, and understand the information needed to make decisions concerning voting and other matters (Rawls 2005; Elliott and Resnik 2019). To promote transparency and openness pertaining to bio-risks, leaders of research institutions must inform community members and the public about proposals for dangerous biological research and risk-management plans so they can decide whether the risks are acceptable (Evans et al. 2015; Yeh et al. 2017; Author 2021; Haines and Gronvall 2023). Collecting and analyzing data on laboratory accidents, security breaches and other adverse outcomes (mentioned earlier) can also help to promote transparency and openness.

Transparency and openness can be difficult to achieve because there is inherent tension between these values and other values, such as protecting the privacy of human research participants, intellectual property, research interests, and proprietary or classified information (Resnik 2006, 2020). However, it is usually possible for scientists and institutional officials to share information the public needs to know without compromising other values. For example, institutional officials can disclose the types of studies that are being proposed without revealing detailed information that could threaten the privacy of research participants or researchers' interests.

Including community members on institutional ethics committees, such as IBCs, IRBs, or ACUCs, helps to promote democracy by giving local people a voice in deliberations about proposed research (Presidential Commission for the Study of Bioethical Issues 2016). However, community representation on ethics committees is not nearly enough to ensure that decision-making related to bio-risks is democratic because important choices must be made long before a study reaches the IBC, such as deciding whether dangerous biological research should be funded and conducted, and how its risks should be managed. While some politicians and concerned citizens have been highly critical about government funding of GOF research, they have not said much about funding other types of research that raise biosafety and biosecurity issues. For example, during his questioning of Dr. Fauci, Senator Paul focused on the funding of GOF research by the NIH and did not address the issue of how many BSL-3 or BSL-4 labs the US needs to study infectious diseases or how safety at these labs can be improved (Basu 2021; National Institutes of Health. 2023).

For an example of deliberative democracy related to building dangerous biological laboratories, consider the controversy concerning $1.25 billion National Bio and Agro-Defense Facility (NBADF), BSL-4 lab located in Manhattan, KS, which opened on May 23, 2023 (Eaves 2020; Associated Press 2023). Following the anthrax attacks of 2001, the US government decided to expand the number of BSL-3 and BLS-4 labs supported by federal agencies to develop countermeasures to bioterrorism (Eaves 2020). The Department of Homeland Security (DHS) initially proposed upgrading a BSL-3 lab on Plum Island, NY to a BSL-4 lab, but abandoned this idea after Senator Hillary Clinton and Representative Timothy Bishop opposed it (Eaves 2020). The DHS considered several sites for the NBADF but selected Manhattan because of its remoteness from heavily populated areas and its proximity to Kansas State University.^13^ Also, Kansas politicians, such as Senator Pat Roberts, encouraged DHS officials to build the NBADF in Manhattan because it would create 400 jobs generate hundreds of millions of dollars each year for the local economy (Eaves 2020). Although elected officials played key roles in the selection of the site for the NBADF, it is not clear that the decisions they made reflected the will of the people. Many Manhattan residents strongly opposed construction NBADF because they feared that a lab accident could have devastating impacts on agriculture, especially livestock. While NBADF critics were able to express their views at public meetings held by DHS and communicate their opposition to elected officials, it is unclear whether their concerns had a significant impact on DHS’ decision, other than to delay it (Eaves 2020).

Another example of democratic oversight of bio-risks occurred in the State of Florida from 2009 to 2022 (Resnik 2019). In 2009, the Florida Keys Mosquito Control District (FKMCD) approached Oxitec about conducting a field trial of its GM *Aedes aegypti* mosquitoes. Oxitec had developed GM male mosquitoes (which do not bite) with a genetic variant rendering their offspring incapable of reproducing because they die before reaching maturity unless they are dosed with the antibiotic tetracycline. Oxitec had already conducted fields trials in the Cayman Islands and Brazil. Studies had shown the Oxitec’s mosquitoes can cause *Aedes aegypti* populations to decline by over 90% (Carvalho et al. 2015). *Aedes aegypti* mosquitoes carry several diseases, including dengue, Zika, and Yellow Fever.

From 2012 to 2014, while Oxitec’s application was under review at the US Department of Agriculture (USDA) and then at the FDA, the FKMCD polled residents about a proposed field trial in Key Haven, FL and held public meetings about it. Although participants in these meetings expressed serious concerns about the environmental and public health impacts of the field trial, initial polling showed most members of the local community supported it (Resnik 2018; Schairer et al 2021). However, environmental groups soon launched a media campaign against the field trial and asked people to communicate their opposition by submitting public comment to the FDA. Florida resident Milagros Demier sent a petition opposing the field trial to the FDA, which was signed by 165,000 people, more than four times the population of Key Haven (Schairer et al 2021). In the spring of 2016, Oxitec received permission from the FDA to conduct a field trial in Key Haven. Later that year, Florida voters approved a referendum for GM mosquito field trials in the state of Florida and Monroe County voters approved a referendum for field trials in their county, but Key Haven voters did not approve the field trials, so the FKMCD and Oxitec decided to conduct the trials elsewhere. In 2021, following a public comment period, the EPA, which had taken over federal jurisdiction of GM mosquitoes, gave Oxitec permission to conduct field trials in Cudjoe Key, Ramrod Key, and Vaca Key, Florida, and in Harris County, Texas and Tulare County, CA (Oxitec 2022). In April 2022, Oxitec announced that the lethal gene was successfully transmitted to female mosquitoes in the Florida Keys field trials and that none of them reached adulthood. The study was a small, pilot project that was not designed to test efficacy (Waltz 2022).

One could argue that the events which transpired in Florida represent a paradigmatic case of procedurally fair, democratic decision-making related to the oversight of bio-risks. The company sought permission for its studies from the relevant governmental authorities and citizens had an opportunity to express their opinions through voting and by petitioning elected and appointed officials. Citizens of Key Haven were not forced to accept bio-risks they did not approve of, and Oxitec was able to obtain approval for field trials in places where there was not strong community opposition.

However, some commentators have argued that the democratic process in this case was not fair or effective (Meghani and Kuzma 2018). First, there was a lack of clear regulatory guidance concerning approval of the GM mosquito trials, because three federal agencies took turns claiming jurisdiction (Schairer et al 2021). Lack of clear regulatory guidance was a problem not only for Oxitec and the FKMCD but also for those seeking to petition the government about the proposed field trials. Although the FDA received 2649 comments and the EPA received 500, it is not clear how the agencies incorporated these comments into their decision-making (Schairer et al 2021). Second, because Oxitec and environmental groups had exerted substantial influence on government officials and the public, there is some doubt as to whether the votes that were taken reflected the will of the people or corporate or political interests (Meghani and Kuzma 2018). It is worth noting that public opinion about the field trials shifted from positive to negative after environmental groups inserted themselves into this controversy (Resnik 2018). Third, the process that could have played a key role in soliciting and forming public opinion in a fair and effective way, i.e., community engagement, was far from ideal (Schairer et al 2021). FKMCD’s “engagement” was a one-way sharing of scientific and technical information rather than a two-way dialogue. To be fair and effective, community/public engagement should promote open discussion in which parties on different sides of an issue share information, concerns, and values and try to understand each other’s point of view. Engagement should be respectful, meaningful, and inclusive (Lavery et al. 2010; Tindana et al. 2015; National Academies of Sciences, Engineering, and Medicine 2016; Conley et al. 2023).

As one can see from these cases, there is a pressing need for more critical reflection and empirical research on how to meaningfully involve communities and the public in decision-making related to the assessment, management, and distribution of bio-risks (Schoch-Spana et al. 2017). Bioethicists can contribute to this discussion by examining moral concepts, principles, and practices related to community and public engagement.

## Conclusion

Biosafety (and to a lesser extent biosecurity) tends to be viewed as a technical matter best addressed by scientific and public health experts. This article has shown, contrarily, that biosafety raises important ethical issues related to the assessment, management, and distribution of bio-risks, and that biosafety practice, education, and policy could therefore benefit from incorporating insights from bioethics. Some topics where bioethics may make important contributions to biosafety (and biosecurity) include:

- Weighing the risks and benefits and benefits of biological research.
- Contributing to deliberations about managing the risks of biological research; such as deciding which BSL level is appropriate for a study.
- Fostering a safety-oriented culture in a biolab or research institute.
- Incorporating biosafety and biosecurity issues into education and training in the responsible conduct of research.
- Thinking about what is means for laboratory workers to make informed, voluntary choices concerning participation in dangerous biological research and potential barriers to effective consent, such as the outsourcing of dangerous biological research to low-income countries.
- Reflecting on the role of democratic processes and principles, such as community and public engagement, transparent/open communication, and public membership on research committees, in oversight of bio-risks.
- Examining inherent conflicts between transparency/openness in science and other important values, such as protection of the privacy of human subjects, intellectual property, and proprietary and classified information, and how to resolve these conflicts.
- Developing codes of conduct related to biosafety and biosecurity.

There are probably other topics where bioethics can make contributions to biosafety that have not been mentioned above. Hopefully, the conclusions and proposals defended in this article will stimulate further discussion of the bioethics of biosafety.

## Data Availability

No data availability

## Footnotes

1 Genetic engineering involves the direct modification of an organism’s genome; for example, using a molecular tool, such as CRISPR, to insert a DNA sequence into a virus that enables it to produce a protein that help it infect human cells (Menachery et al. 2015). Serial passaging is a form of experimentation in which one infects animals with a pathogen and then inoculates other animals with secretions or other biomaterials from the original hosts, based on signs or symptoms they display, such as degree of illness. Passaging is a form of directed evolution (Russell et al. 2012; Sirotkin and Sirotkin 2020).

2 Evans et al. (2015), Selgelid (2016), Resnik (2021), and Johnson (2024) are noteworthy exceptions to this generalization.

3 In this paper I will use the technical, laboratory definition of biosafety from DHHS. Biosafety is sometimes conceived of more broadly as protecting the population from biological risks, but I will not use this definition because it does not clearly distinguish between biosafety from biosecurity.

4 While the public has some control over funding decisions made by government agencies, such as the NIH and CDC, it has almost no control over funding decisions made by private companies (Resnik 2007). However, the public can influence private decisions by supporting laws and regulations that incentivize or disincentive companies concerning specific courses of action. For example, the National Childhood Vaccine Injury Act (1986) absolves vaccine manufacturers from liability for injuries resulting from the administration of vaccines. The law was passed to provide companies with economic incentives to invest in vaccine research and development because it greatly reduces the legal costs associated with marketing vaccines.

5 Some authors distinguish between ‘ethical’ and ‘moral’ but I will use the two words interchangeably in this article.

6 A notable exception is recent work on global catastrophic biological risks, which discusses the importance of taking global action to address biosafety, biosecurity, and other biological risks. See Schoch-Spana et al. (2017), Kambouris (2023), and Johnson (2024).

7 A gene drive system is a molecular mechanism that increases the prevalence of a gene in a population, irrespective of its effects on organismic fitness.

8 A hazard is something that can produce harm. A risk is the probability that a harm will occur. Dangerous biological agents and toxins can be viewed as hazardous biological materials that pose a risk of harm.

9 One might also argue that employers have obligations to take reasonably measures to promote safety in the workplace (Resnik 2012).

10 For a person’s consent to be valid, their decision must free from coercion or undue influence, and they must have sufficient information to make a reasonable choice. Additionally, the person must be capable of making autonomous choices.

11 The role of non-publicly-accountable experts and bureaucrats in government decision-making raises profound moral and political issues for modern democracies that will not be addressed here. See Brown (2009), Lofgren (2016), and Bertsou and Caramani (2021).

12 Engagement may serve purposes other than democratic governance, such as promoting trust between scientists and the community or public, building partnerships, and securing “buy in” or support for a particular project or technology (National Academies of Sciences, Engineering, and Medicine 2016; Kolopack and Lavery 2017; Neuhaus 2018; Borthwick et al. 2023).

13 It is worth noting that the WIV is located in Wuhan, a large urban center with over 12 million residents.

## References

[CR1] Associated Press. 2023. After years of controversy, national bio-defense lab opens in Kansas. Associated Press, May 24, 2023. https://apnews.com/article/national-bio-lab-manhattan-kansas-opening-5fb349d58192d6821d4efa5d5a80731b

[CR2] American Association for the Accreditation of Laboratory Animal Care International. 2023. https://www.aaalac.org/

[CR3] American Biosafety Association International. 2023. Who we are. https://absa.org/about/

[CR4] American Lung Association. 2023. Severe Acute Respiratory Syndrome (SARS). https://www.lung.org/lung-health-diseases/lung-disease-lookup/severe-acute-respiratory-syndrome-sars

[CR5] Andersen, K.G., A. Rambaut, W.I. Lipkin, E.C. Holmes, and R.F. Garry. 2020. The proximal origin of SARS-CoV-2. *Nature Medicine* 26: 450–452. 10.1038/s41591-020-0820-9 PMC7095063 32284615

[CR6] Antes, A.L., A. Kuykendall, and J.M. DuBois. 2019. The lab management practices of “research exemplars” that foster research rigor and regulatory compliance: A qualitative study of successful principal investigators. *PLoS One* 14 (4): e0214595. 31017929 10.1371/journal.pone.0214595 PMC6481787

[CR7] Barry, B. 1991. *Theories of Justice*. Berkeley: University of California Press.

[CR8] Basu Z. 2021. Fauci and Rand Paul clash over NIH funding for Wuhan Institute of Virology. Axios, May 12, 2021. https://www.axios.com/2021/05/11/fauci-rand-paul-wuhan-lab-coronavirus

[CR9] Beeckman, D.S., and P. Rüdelsheim. 2020. Biosafety and biosecurity in containment: A regulatory overview. *Frontiers in Bioengineering and Biotechnology* 8: 650. 32719780 10.3389/fbioe.2020.00650 PMC7348994

[CR10] Bender, F.F. 2007. “I’m sorry” laws and medical liability. *Virtual Mentor* 9 (4): 300–304. 23217974 10.1001/virtualmentor.2007.9.4.hlaw1-0704

[CR11] Berg, P., D. Baltimore, H.W. Boyer, S.N. Cohen, R.W. Davis, D.S. Hogness, D. Nathans, R. Roblin, J.D. Watson, S. Weissman, and N.D. Zinder. 1974. Letter: Potential biohazards of recombinant DNA molecules. *Science* 185 (4148): 303. 10.1126/science.185.4148.303 4600381

[CR12] Berg, P., D. Baltimore, S. Brenner, R.O. Roblin, and M.F. Singer. 1975. Summary statement of the Asilomar conference on recombinant DNA molecules. *Proceedings of National Academy of Sciences USA* 72 (6): 1981–1984. 10.1073/pnas.72.6.1981 PMC432675 806076

[CR13] Bertsou, E., and D. Caramani, eds. 2021. *The Technocratic Challenge to Democracy*. New York: Routledge.

[CR14] Borthwick, J., N. Evertsz, and B. Pratt. 2023. How should communities be meaningfully engaged (if at all) when setting priorities for biomedical research? Perspectives from the biomedical research community. *BMC Medical Ethics* 24: 6. 36747191 10.1186/s12910-022-00879-5 PMC9900561

[CR15] Brown, M.B. 2009. *Science in Democracy: Expertise, Institutions, and Representation*. Cambridge: MIT Press. 10.1007/s11024-011-9179-x PMC3167039 21957318

[CR16] Butler, D. 2009. European biosafety labs set to grow. *Nature* 462: 146–147. 19907462 10.1038/462146a PMC7094912

[CR17] Calvert, J., and G. Arbuthnutt. 2023. What really happened inside the Wuhan lab weeks before Covid erupted. *The Times*, June 10, 2023. https://www.thetimes.co.uk/article/inside-wuhan-lab-covid-pandemic-china-america-qhjwwwvm0. Accessed 10 June 2023.

[CR18] Cartagena Protocol on Biosafety. 2023. https://bch.cbd.int/protocol/

[CR19] Carvalho, D.O., A.R. McKemey, L. Garziera, R. Lacroix, C.A. Donnelly, L. Alphey, A. Malavasi, and M.L. Capurro. 2015. Suppression of a field population of Aedes aegypti in Brazil by sustained release of transgenic male mosquitoes. *PLoS Neglected Tropical Diseases* 9 (7): e0003864. 26135160 10.1371/journal.pntd.0003864 PMC4489809

[CR20] Casadevall, A., D. Howard, and M.J. Imperiale. 2014. An epistemological perspective on the value of gain-of-function experiments involving pathogens with pandemic potential. *Bio* 5 (5): e01875-14. 10.1128/mBio.01875-14 PMC4172079 25227471

[CR21] Cello, J., A. Paul, and E. Wimmer. 2002. Chemical synthesis of poliovirus cDNA: Generation of infectious virus in the absence of natural template. *Science* 297: 1016–1018. 12114528 10.1126/science.1072266

[CR22] Centers for Disease Control and Prevention. 2015. CDC releases report on lab incident. https://www.cdc.gov/media/releases/2015/s0204-ebola-lab.html

[CR23] Centers for Disease Control and Prevention. 2018. History of 1918 Flu Pandemic. https://www.cdc.gov/flu/pandemic-resources/1918-commemoration/1918-pandemic-history.htm

[CR24] Centers for Disease Control and Prevention. 2020. Biosafety in Microbiological and Biomedical Laboratories, 6th ed. https://www.cdc.gov/labs/BMBL.html

[CR25] Centers for Disease Control and Prevention. 2021. Zoonotic diseases. https://www.cdc.gov/onehealth/basics/zoonotic-diseases.html

[CR26] Chan, A. 2024. Why the pandemic probably started in a lab: 5 key points. *New York Times*. https://www.nytimes.com/interactive/2024/06/03/opinion/covid-lab-leak.html. Accessed 3 June 2024.

[CR27] Christiano, T., and S. Bajaj. 2022. Democracy. Stanford Encyclopedia of Ethics. https://plato.stanford.edu/entries/democracy/

[CR28] CNN. 2023. 2001 anthrax attacks: fast facts. https://www.cnn.com/2013/08/23/health/anthrax-fast-facts/index.html

[CR29] Cohen D. 2023. China continues to block efforts to determine Covid’s origins, lawmakers say. Politico. https://www.politico.com/news/2023/03/05/covid-origin-theories-china-00085546. Accessed 5 Mrach 2023.

[CR30] European Commission. 2000. Communication from the Commission on the precautionary principle https://eur-lex.europa.eu/legal-content/EN/TXT/?uri=celex%3A52000DC0001

[CR31] Senate Committee on Health Education, Labor, and Pension. 2022. An Analysis of the Origins of the COVID-19 Pandemic. https://www.help.senate.gov/imo/media/doc/report_an_analysis_of_the_origins_of_covid-19_102722.pdf

[CR32] Conley, J.M., R.J. Cadigan, A.M. Davis, E.T. Juengst, K. Kuczynski, R. Major, H. Stancil, J. Villa-Palomino, M. Waltz, and G.E. Henderson. 2023. The promise and reality of public engagement in the governance of human genome editing research. *The American Journal of Bioethics* 23 (7): 9–16. 10.1080/15265161.2023.2207502 PMC10367578 37204137

[CR33] Connell, N. 2011. Biological agents in the laboratory: The regulatory issues. *Public Interest Report, Fall* 2011: 1–6.

[CR34] Cornwall, W. 2023. New U.S. lab will work with deadly animal pathogens—in the middle of farm country. Science. https://www.science.org/content/article/new-u-s-lab-will-work-deadly-animal-pathogen-middle-farm-country. Accessed 23 May 2023.

[CR35] Crichton M. 1969. The Andromeda Strain. New York, NY: Alfred A Knopf.

[CR36] Cyranoski, D. 2017. Inside the Chinese lab poised to study world’s most dangerous pathogens. *Nature* 542: 399–400. 28230144 10.1038/nature.2017.21487

[CR37] Davis, C.T., L.M. Chen, C. Pappas, J. Stevens, T.M. Tumpey, L.V. Gubareva, J.M. Katz, J.M. Villanueva, R.O. Donis, and N.J. Cox. 2014. Use of highly pathogenic avian influenza A(H5N1) gain-of-function studies for molecular-based surveillance and pandemic preparedness. *mBio* 5 (6): e02431-14. 25505125 10.1128/mBio.02431-14 PMC4278543

[CR38] Department of Health and Human Services. 2015. Biosecurity. https://www.phe.gov/s3/BioriskManagement/biosecurity/Pages/default.aspx 10.3109/15360288.2015.1037530 26095483

[CR39] Department of Health and Human Services. 2017a. Biosafety. https://www.phe.gov/s3/BioriskManagement/biosafety/Pages/default.aspx

[CR40] Department of Health and Human Services. 2017b. Framework for guiding funding decisions about proposed research involving enhanced potential pandemic pathogens. Available at: https://www.phe.gov/s3/dualuse/Documents/P3CO.pdf

[CR41] Department of Health and Human Services. 2021. Dual use research of concern. https://www.phe.gov/s3/dualuse/Pages/default.aspx

[CR42] Director of National Intelligence. 2023. Updated assessment on COVID-19 origins. https://www.dni.gov/files/ODNI/documents/assessments/Declassified-Assessment-on-COVID-19-Origins.pdf

[CR43] DiRita, V., and S. Bertuzzi. 2021. Gain-of-function research advances knowledge and saves lives. *Stat News*. https://www.statnews.com/2021/12/23/gain-of-function-research-advances-knowledge-and-saves-lives/. Accessed 23 December 2021.

[CR44] Dover, N., J.R. Barash, K.K. Hill, G. Xie, and S.S. Arnon. 2014. Molecular characterization of a novel botulinum neurotoxin type H gene. *Journal of Infectious Disease* 209 (2): 192–202. 10.1093/infdis/jit450 24106295

[CR45] Eaves, E. 2020. The risks of building too many bio labs. *The New Yorker*, March 18, 2020. https://www.newyorker.com/science/elements/the-risks-of-building-too-many-bio-labs. Accessed 18 March 2020.

[CR46] Elliott, K.C., and D.B. Resnik. 2019. Making open science work for science and society. *Environmental Health Perspectives* 127 (7): 75002. 31353949 10.1289/EHP4808 PMC6792383

[CR47] Evans, N.G., M. Lipsitch, and M. Levinson. 2015. The ethics of biosafety considerations in gain-of-function research resulting in the creation of potential pandemic pathogens. *Journal of Medical Ethics* 41 (11): 901–908. 26320212 10.1136/medethics-2014-102619 PMC4623968

[CR48] Evans, N.G., M.J. Selgelid, and M.R. Simpson. 2022. Reconciling regulation with scientific autonomy in dual-use research. *Journal of Medicine and Philosophy* 47 (1): 72–94. 35137173 10.1093/jmp/jhab041

[CR49] Fauci, A.S. 2012. Research on highly pathogenic H5N1 influenza virus: the way forward. *mBio* 3 (5): e00359-12. 23047751 10.1128/mBio.00359-12 PMC3484390

[CR50] Feeser, V.R., A.K. Jackson, N.M. Savage, T.A. Layng, R.K. Senn, H.S. Dhindsa, S.A. Santen, and R.R. Hemphill. 2021. When safety event reporting is seen as punitive: “I’ve been PSN-ed!” *Annals of Emergency Medicine* 77 (4): 449–458. 32807540 10.1016/j.annemergmed.2020.06.048

[CR51] Fishkin, J.S. 2011. *When the People Speak: Deliberative Democracy and Public Consultation*. Oxford: Oxford University Press.

[CR52] Fouchier, R.A. 2015. Studies on influenza virus transmission between ferrets: the public health risks revisited. *mBio* 6 (1): e02560-14. 25616377 10.1128/mBio.02560-14 PMC4323420

[CR53] Fouchier, R.A., A. García-Sastre, and Y. Kawaoka. 2012. The pause on Avian H5N1 influenza virus transmission research should be ended. *mBio* 3 (5): e00358-12. 23047750 10.1128/mBio.00358-12 PMC3484389

[CR54] Frischknecht, F. 2003. The history of biological warfare. *EMBO Reports* 4 (Suppl 1): S47-52. 12789407 10.1038/sj.embor.embor849 PMC1326439

[CR55] Gettayacamin, M., and L. Retnam. 2017. AAALAC International standards and accreditation process. *Toxicological Research* 33 (3): 183–189. 28744349 10.5487/TR.2017.33.3.183 PMC5523556

[CR56] Gilbert, N. 2023. China Initiative’s shadow looms large for US scientists. *Nature* 615: 198–199. 36829062 10.1038/d41586-023-00543-x

[CR57] Gronvall, G., and T.K. Sell. 2022. Opinion: Destroy all samples of the smallpox virus. The Scientist, June 1, 2022. https://www.the-scientist.com/news-opinion/opinion-destroy-all-samples-of-the-smallpox-virus-70088. Accessed 1 June 2022.

[CR58] Gutmann, A., and D. Thompson. 1998. *Democracy and Disagreement*. Cambridge: Harvard University Press.

[CR59] Gutmann, A., and D. Thompson. 2004. *Why Deliberative Democracy?* Princeton: Princeton University Press.

[CR60] Haider, N., P. Rothman-Ostrow, A.Y. Osman, L.B. Arruda, L. Macfarlane-Berry, L. Elton, M.J. Thomason, D. Yeboah-Manu, R. Ansumana, N. Kapata, L. Mboera, J. Rushton, T.D. McHugh, D.L. Heymann, A. Zumla, and R.A. Kock. 2020. COVID-19: Zoonosis or emerging infectious disease? *Frontiers in Public Health* 8: 596944. 33324602 10.3389/fpubh.2020.596944 PMC7725765

[CR61] Haines, C.A., and G.K. Gronvall. 2023. Improving U.S. biosafety and biosecurity: Revisiting recommendations from the federal experts security advisory panel and the Fast Track Action Committee on Select Agent Regulations. *Applied Biosafety* 28 (1): 43–54. 36895583 10.1089/apb.2022.0025 PMC9991423

[CR62] Harrison, N.L., and J.D. Sachs. 2022. A call for an independent inquiry into the origin of the SARS-CoV-2 virus. *Proceedings of the National Academy of Sciences USA* 119 (21): e2202769119. 10.1073/pnas.2202769119 PMC9173817 35588448

[CR63] Himmel, M. 2023. *Biosecurity Risk Assessment in the Life Sciences*. Solna: Stockholm International Peace Research Institute.

[CR64] Holm, S. 2017. The bioethicist who cried “synthetic biology”: An analysis of the function of bioterrorism predictions in bioethics. *Quarterly of Healthcare Ethics* 26: 230–238. 10.1017/S0963180116000827 28361720

[CR65] Imai, M., T. Watanabe, M. Hatta, S.C. Das, M. Ozawa, K. Shinya, G. Zhong, A. Hanson, H. Katsura, S. Watanabe, C. Li, E. Kawakami, S. Yamada, M. Kiso, Y. Suzuki, E.A. Maher, G. Neumann, and Y. Kawaoka. 2012. Experimental adaptation of an influenza H5 HA confers respiratory droplet transmission to a reassortant H5 HA/H1N1 virus in ferrets. *Nature* 486 (7403): 420–428. 22722205 10.1038/nature10831 PMC3388103

[CR66] Institute of Medicine. 2000. *To Err Is Human: Building a Safer Health System*. Washington: National Academies Press. 25077248

[CR67] International Federation of Biosafety Associations. 2023. Who we are. https://internationalbiosafety.org/who-we-are/

[CR68] Jackson, D.A., R.H. Symons, and P. Berg. 1972. Biochemical method for inserting new genetic information into DNA of Simian Virus 40: Circular SV40 DNA molecules containing lambda phage genes and the galactose operon of Escherichia coli. *Proceedings of the National Academy of Sciences USA* 69 (10): 2904–2909. 10.1073/pnas.69.10.2904 PMC389671 4342968

[CR69] Jacobsen, R. 2021. Inside the risky bat-virus engineering that links America to Wuhan. MIT Technology Review. https://www.technologyreview.com/2021/06/29/1027290/gain-of-function-risky-bat-virus-engineering-links-america-to-wuhan/. Accessed 29 June 2021.

[CR70] Johnson, T.F. 2024. For the good of the globe: moral reasons for states to mitigate global catastrophic biological risks. *Bioethical Inquiry*. 10.1007/s11673-024-10337-z. 10.1007/s11673-024-10337-z PMC11652646 38329644

[CR71] Kaebnick, G.E., E. Heitman, J.P. Collins, J.A. Delborne, W.G. Landis, K. Sawyer, L.A. Taneyhill, and D.E. Winickoff. 2016. Precaution and governance of emerging technologies. *Science* 354 (6313): 710–711. 27846595 10.1126/science.aah5125

[CR72] Kaiser J. (2024). White House overhauls rules for risky pathogen studies. Science. https://www.science.org/content/article/white-house-overhauls-rules-risky-pathogen-studies?utm_source=sfmc&utm_medium=email&utm_content=alert&utm_campaign=SCIeToc&et_rid=35371335&et_cid=5207966. Accessed 7 May 2024. 10.1126/science.adq3094 38723070

[CR73] Kambouris, M.E. 2023. Global catastrophic biological risks in the post-COVID-19 world: Time to act is now. *OMICS* 27 (4): 153–170. 36946656 10.1089/omi.2022.0178

[CR74] Kimman, T.G., E. Smit, and M.R. Klein. 2008. Evidence-based biosafety: A review of the principles and effectiveness of microbiological containment measures. *Clinical Microbiology Review* 21 (3): 403–25. 10.1128/CMR.00014-08 PMC2493080 18625678

[CR75] Klotz, L.C. 2015. Comments on Fouchier’s calculation of risk and elapsed time for escape of a laboratory-acquired infection from his laboratory. *mBio* 6 (2): e00268-15. 25873376 10.1128/mBio.00268-15 PMC4453553

[CR76] Kolopack, P.A., and J.V. Lavery. 2017. Informed consent in field trials of gene-drive mosquitoes. *Gates Open Research* 1: 14. 29355214 10.12688/gatesopenres.12771.1 PMC5757819

[CR77] Kuhlau, F., A.T. Höglund, K. Evers, and S. Eriksson. 2011. A precautionary principle for dual use research in the life sciences. *Bioethics* 25 (1): 1–8. 10.1111/j.1467-8519.2009.01740.x 19594724

[CR78] Lamont, J. 2017. Distributive justice. Stanford Encyclopedia of Philosophy. https://plato.stanford.edu/entries/justice-distributive/

[CR79] Lavery, J.V., P.O. Tinadana, T.W. Scott, L.C. Harrington, J.M. Ramsey, C. Ytuarte-Nuñez, and A.A. James. 2010. Towards a framework for community engagement in global health research. *Trends in Parasitology* 26 (6): 279–83. 20299285 10.1016/j.pt.2010.02.009

[CR80] Leitenberg, M., and R.A. Zilinskas. 1989. *The Soviet Biological Weapons Program: A History*. Cambridge: Harvard University Press.

[CR81] Lenharo, M. 2024. US halts funding to controversial virus-hunting group: what researchers think. Nature 16 May. https://www.nature.com/articles/d41586-024-01460-3. Accessed 16 May 2024. 10.1038/d41586-024-01460-3 38755306

[CR82] Lewis, T. 2023. What new evidence from the Wuhan Market tells us about COVID’s origins. Scientific American, April 12, 2023. https://www.scientificamerican.com/article/what-new-evidence-from-the-wuhan-market-tells-us-about-covids-origins1/. Accessed 12 April 2023.

[CR83] Lipsitch, M. 2018. Why do exceptionally dangerous gain-of-function experiments in influenza? *Methods in Molecular Biology* 1836: 589–608. 30151594 10.1007/978-1-4939-8678-1_29 PMC7119956

[CR84] Lipsitch, M., and B.R. Bloom. 2012. Rethinking biosafety in research on potential pandemic pathogens. *mBio* 3 (5): e00360-12. 23047752 10.1128/mBio.00360-12 PMC3484391

[CR85] Lipsitch, M., and T.V. Inglesby. 2014. Moratorium on research intended to create novel potential pandemic pathogens. *mBio* 5 (6): e02366-14. 25505122 10.1128/mBio.02366-14 PMC4271556

[CR86] Lofgren, M. 2016. *The Deep State: The Fall of the Constitution and the Rise of a Shadow Government*. New York: Penguin Books.

[CR87] Mallapatay, S. 2023. COVID-origins study links raccoon dogs to Wuhan market: What scientists think. *Nature* 615: 771–772. 36944776 10.1038/d41586-023-00827-2

[CR88] Marcassoli, A., M. Leonardi, M. Passavanti, V. De Angelis, E. Bentivegna, P. Martelletti, and A. Raggi. 2023. Lessons learned from the lessons learned in public health during the first years of COVID-19 pandemic. *International Journal of Environmental Research and Public Health* 20 (3): 1785. 36767152 10.3390/ijerph20031785 PMC9914715

[CR89] Meghani, Z., and J. Kuzma. 2018. Regulating animals with gene drive systems: Lessons from the regulatory assessment of a genetically engineered mosquito. *Journal of Responsible Innovation* 5 (sup1): S203–S222. 10.1080/23299460.2017.1407912

[CR90] Menachery, V.D., B.L. Yount Jr., K. Debbink, S. Agnihothram, L.E. Gralinski, J.A. Plante, R.L. Graham, T. Scobey, X.Y. Ge, E.F. Donaldson, S.H. Randell, A. Lanzavecchia, W.A. Marasco, Z.L. Shi, and R.S. Baric. 2015. A SARS-like cluster of circulating bat coronaviruses shows potential for human emergence. *Nature Medicine* 21: 1508–1513. 10.1038/nm.3985 PMC4797993 26552008

[CR91] Miller, S., and M.J. Selgelid. 2007. Ethical and philosophical consideration of the dual-use dilemma in the biological sciences. *Science and Engineering Ethics* 13: 523–580. 18060518 10.1007/s11948-007-9043-4 PMC7089176

[CR92] Mourya, D.T., P.D. Yadav, A. Khare, and A.H. Khan. 2017. Certification & validation of biosafety level-2 & biosafety level-3 laboratories in Indian settings & common issues. *Indian Journal of Medical Research* 146 (4): 459–467. 29434059 10.4103/ijmr.IJMR_974_16 PMC5819027

[CR93] National Academies of Sciences, Engineering, and Medicine. 2017. *Fostering Integrity in Research*. Washington: National Academies Press. 29341557

[CR94] National Academies of Sciences, Engineering, and Medicine. 2016. *Gene Drives on the Horizon: Advancing Science, Navigating Uncertainty, and Aligning Research with Public Values*. Washington: National Academies Press. 27536751

[CR95] National Childhood Vaccine Injury Act. 1986. Public Law 99-660. https://www.congress.gov/bill/99th-congress/house-bill/5546

[CR96] National Institutes of Health. 2019. FAQs on Institutional Biosafety Committee (IBC) administration—May 2019. https://osp.od.nih.gov/policies/biosafety-and-biosecurity-policy/faqs-on-institutional-biosafety-committee-ibc-administration-may-2019/

[CR97] National Institutes of Health. 2022. Updated guidance: requirement for instruction in the responsible conduct of research. https://grants.nih.gov/grants/guide/notice-files/NOT-OD-22-055.html

[CR98] National Institutes of Health. 2023. The need for biosafety labs. https://www.niaid.nih.gov/research/biosafety-labs-needed

[CR99] National Research Council. 1989. *Biosafety in the Laboratory: Prudent Practices for the Handling and Disposal of Infectious Materials*. Washington: National Academies Press. 25032323

[CR100] National Research Council. 2004. *Biotechnology Research in the Age of Terrorism*. Washington: National Academies Press. 25057686

[CR101] National Research Council. 2011. *Guide to the Care and Use of Laboratory Animals*, 8th ed. Washington: National Academies Press.

[CR102] National Research Council. 2015. *Potential Risks and Benefits of Gain-of-Function Research: Summary of a Workshop*. Washington: National Academies Press. 25719185

[CR103] National Science Advisory Board for Biosecurity. 2005. Summary of inaugural meeting. https://osp.od.nih.gov/wp-content/uploads/NSABB_Meeting_Minutes_June-July_2005.pdf

[CR104] National Science Advisory Board for Biosecurity. 2014. Minutes. https://osp.od.nih.gov/wp-content/uploads/NSABB_Meeting_Minutes-Oct_2014.pdf. Accessed 22 October 2014.

[CR105] National Science Advisory Board for Biosecurity. 2023. About us. https://osp.od.nih.gov/about-us/

[CR106] Neuhaus, C.P. 2018. Community engagement and field trials of genetically modified insects and animals. *Hastings Cent Rep* 48 (1): 25–36. 10.1002/hast.808 29457234

[CR107] Nuffield Council on Bioethics. 2007. *Public Health: Ethical Issues*. London: Nuffield Council.

[CR108] Oxitec. 2022. U.S. EPA approves Oxitec mosquito pilot projects in California and Florida; press release; March 8, 2022. https://www.oxitec.com/en/news/us-epa-approves-oxitec-mosquito-pilot-projects-in-california-and-florida. Accessed 8 March 2022.

[CR109] Parry, J. 2004. Breaches of safety regulations are probable cause of recent SARS outbreak, WHO says. *BMJ* 328 (7450): 1222. 10.1136/bmj.328.7450.1222-b PMC416634 15155496

[CR110] Perkins, D., K. Danskin, A.E. Rowe, and A.A. Livinski. 2019. The culture of biosafety, biosecurity, and responsible conduct in the life sciences: A comprehensive literature review. *Appl Biosaf* 24 (1): 34–45. 36034634 10.1177/1535676018778538 PMC9093240

[CR111] Presidential Commission for the Study of Bioethical Issues. 2016. *Bioethics for Every Generation: Deliberation and Education in Health, Science, and Technology*. Washington: Presidential Commission.

[CR112] Federal Select Agent Program. 2022. Select agents and toxins. https://www.selectagents.gov/compliance/faq/sat.htm

[CR113] Rappert, B., and M.J. Selgelid, eds. 2013. *On the Dual Uses of Science and Ethics: Principles, Practices, and Prospects*. Canberra: Australian National University.

[CR114] Rawls, J. 1971. *A Theory of Justice*. Cambridge: Harvard University Press.

[CR115] Rawls, J. 2005. *Political Liberalism*, 2nd ed. New York: Columbia University Press.

[CR116] Relman, D.A. 2014. “Inconvenient truths” in the pursuit of scientific knowledge and public health. *Journal of Infectious Diseases* 209 (2): 170–172. 24106297 10.1093/infdis/jit529 PMC3873791

[CR154] Resnik, D.B. 2004. Owning the Genome: A Moral Analysis of DNA Patenting. Albany, NY: SUNY Press.

[CR160] Resnik, D.B. 2006. Openness vs. secrecy in scientific research. Episteme 2: 135–147. 10.3366/epi.2005.2.3.135 PMC2991133 21113394

[CR162] Resnik, D.B. 2007. The Price of Truth: How Money Affects the Norms of Science. New York: Oxford University Press.

[CR157] Resnik, D.B. 2012. Environmental Health Ethics. Cambridge, UK: Cambridge University Press.

[CR155] Resnik, D.B. 2013. H5N1 avian flu research and the ethics of knowledge. Hastings Center Report 43(2): 22–33. 10.1002/hast.143 PMC3953619 23390001

[CR158] Resnik. D.B. 2014. Ethical issues in field trials of genetically modified disease‐resistant mosquitoes. Developing World Bioethics 14(1): 37–46. 10.1111/dewb.12011 PMC3620724 23279283

[CR159] Resnik, D.B. 2018. Ethics of community engagement in field trials of genetically modified mosquitoes. Developing World Bioethics 18(2):135–143. 10.1111/dewb.12147 PMC12088697 28470752

[CR161] Resnik, D.B. 2019. Two unresolved issues in community engagement for field trials of genetically modified mosquitoes. Pathogens and Global Health 113(5): 238–245. 10.1080/20477724.2019.1670490 PMC6882470 31549925

[CR156] Resnik, D.B. 2020. Balancing transparency and security--ethical consideration. Presentation given the National Science Advisory Board for Biosecurity, January 23, 2020.

[CR152] Resnik, DB. 2021. Precautionary Reasoning in Environmental and Public Health Policy. Cham, Switzerland: Springer.

[CR117] Ridley, M., and E. Chan. 2021. *Viral: The Search for the Origins of COVID-19*. New York: Harper.

[CR118] Rosengard, A., Y. Liu, Z. Nie, and R. Jimenez. 2002. Variola virus immune evasion design: Expression of a highly efficient inhibitor of human complement. *PNAS* 99: 8808–8813. 12034872 10.1073/pnas.112220499 PMC124380

[CR119] Russell, C.A., J.M. Fonville, A.E. Brown, D.F. Burke, D.L. Smith, S.L. James, S. Herfst, S. van Boheemen, M. Linster, E.J. Schrauwen, L. Katzelnick, A. Mosterín, T. Kuiken, E. Maher, G. Neumann, A.D. Osterhaus, Y. Kawaoka, R.A. Fouchier, and D.J. Smith. 2012. The potential for respiratory droplet-transmissible A/H5N1 influenza virus to evolve in a mammalian host. *Science* 336: 1541–7. 22723414 10.1126/science.1222526 PMC3426314

[CR120] Occupational Safety and Health Administration. 2011. Laboratory Safety Guidance. https://www.osha.gov/sites/default/files/publications/OSHA3404laboratory-safety-guidance.pdf

[CR121] Schairer, C.E., J. Najera, A.A. James, O.S. Akbari, and C.S. Bloss. 2021. Oxitec and MosquitoMate in the United States: Lessons for the future of gene drive mosquito control. *Pathogens and Global Health* 115 (6): 365–376. 34313556 10.1080/20477724.2021.1919378 PMC8592615

[CR122] Schneider, B., M.G. Ehrhart, and W.H. Macey. 2013. Organizational climate and culture. *Annual Review of Psychology* 64 (1): 361–388. 10.1146/annurev-psych-113011-143809 22856467

[CR123] Schoch-Spana, M., A. Cicero, A. Adalja, G. Gronvall, T. Kirk Sell, D. Meyer, J.B. Nuzzo, S. Ravi, M.P. Shearer, E. Toner, C. Watson, M. Watson, and T. Inglesby. 2017. Global catastrophic biological risks: Toward a working definition. *Health Security* 15 (4): 323–328. 28745924 10.1089/hs.2017.0038 PMC5576209

[CR124] Schroeder, D., J. Cook, F. Hirsch, S. Fenet, and V. Muthuswamy, eds. 2017. *Ethics Dumping: Case Studies from North-South Research Collaborations*. New York: Springer.

[CR153] Shamoo, A.E. and D.B. Resnik. 2022. Responsible Conduct of Research, 4th ed. New York: Oxford University Press.

[CR125] National Science and Technology Council. 2022. Evidence-based laboratory biorisk management science and technology roadmap. https://www.whitehouse.gov/ostp/news-updates/2022/04/30/nstc-evidence-based-laboratory-biorisk-management-science-technology-roadmap/

[CR126] Selgelid, M.J. 2004. Bioterrorism and smallpox planning: Information and voluntary vaccination. *Journal of Medical Ethics* 30 (6): 558–560. 15574444 10.1136/jme.2003.004176 PMC1733965

[CR127] Selgelid, M.J. 2016. Gain-of-function research: Ethical analysis. *Science and Engineering Ethics* 22 (4): 923–964. 27502512 10.1007/s11948-016-9810-1 PMC4996883

[CR128] Shah, S. 2006. *The Body Hunters: Testing New Drugs on the World’s Poorest Patients*. New York: The New Press.

[CR129] Shinomiya, N., J. Minari, G. Yoshizawa, M. Dando, and L. Shang. 2022. Reconsidering the need for gain-of-function research on enhanced potential pandemic pathogens in the post-COVID-19 era. *Frontiers in Bioengineering and Biotechnology* 10: 966586. 36091454 10.3389/fbioe.2022.966586 PMC9458934

[CR130] Silver, A. 2022. Why the world has no universal biosafety standards. *BMJ* 377: o954. 35422433 10.1136/bmj.o954

[CR131] Sirotkin, K., and D. Sirotkin. 2020. Might SARS-CoV-2 have arisen via serial passage through an animal host or cell culture?: A potential explanation for much of the novel coronavirus’ distinctive genome. *Bioessays* 42 (10): e2000091. 32786014 10.1002/bies.202000091 PMC7435492

[CR132] Stolberg, S., and N. Weiland. 2023. Experts See Lessons for Next Pandemic as Covid Emergency Comes to an End. *New York Times*. https://www.nytimes.com/2023/05/11/us/politics/covid-response-lessons.html. Accessed 11 May 2023.

[CR133] Tindana, P., J. de Vries, M. Campbell, K. Littler, J. Seeley, P. Marshall, J. Troyer, M. Ogundipe, V.P. Alibu, A. Yakubu, M. Parker, as members of the H3A Working Group on Ethics. 2015. Community engagement strategies for genomic studies in Africa: a review of the literature. *BMC Medical Ethics* 12 (16): 24. 10.1186/s12910-015-0014-z PMC4407431 25889051

[CR134] Tumpey, T.M., C.F. Basler, P.V. Aguilar, H. Zeng, A. Solórzano, D.E. Swayne, N.J. Cox, J.M. Katz, J.K. Taubenberger, P. Palese, and A. García-Sastre. 2005. Characterization of the reconstructed 1918 Spanish influenza pandemic virus. *Science* 310 (5745): 77–80. 16210530 10.1126/science.1119392

[CR135] United States Government. 2012. Policy for ocersight of life sciences dual use research of Concern. https://www.phe.gov/s3/dualuse/Documents/us-policy-durc-032812.pdf

[CR136] Wade, N. 2021. The origin of COVID: Did people or nature open Pandora’s box at Wuhan? Bulletin of Atomic Scientists. https://thebulletin.org/2021/05/the-origin-of-covid-did-people-or-nature-open-pandoras-box-at-wuhan/. Accessed 5 May 2021.

[CR137] Wain-Hobson, S. 2013. H5N1 viral-engineering dangers will not go away. *Nature* 495: 411. 23538789 10.1038/495411a

[CR138] Walgate, R. 2004. SARS escaped Beijing lab twice. *Genome Biology* 27: 4.

[CR139] Waltz, E. 2022. Biotech firm announces results from first US trial of genetically modified mosquitoes. *Nature* 604: 608–609. 35437323 10.1038/d41586-022-01070-x

[CR140] Wein, L., and Y. Liu. 2005. Analyzing a bioterror attack on the food supply: The case of botulinum toxin in milk. *Proceedings of the National Academy of Sciences USA* 102: 9984–9989. 10.1073/pnas.0408526102 PMC1161865 15985558

[CR141] Weinstein, R.S. 2011. Should remaining stockpiles of smallpox virus (variola) be destroyed? *Emerging Infectious Diseases* 17 (4): 681–683. 21470459 10.3201/eid1704.101865 PMC3377425

[CR142] The Whitehouse. (2024). Implementation Guidance for the United States Government Policy for Oversight of Dual Use Research of Concern and Pathogens with Enhanced Pandemic Potential. https://www.whitehouse.gov/wp-content/uploads/2024/05/USG-DURC-PEPP-Implementation-Guidance.pdf

[CR143] World Health Organization. 2010. Responsible Life Sciences Research for Global Health Security. https://apps.who.int/iris/handle/10665/70507 26180872

[CR144] World Health Organization. 2017. WHO consultative meeting high/maximum containment (biosafety level 4) laboratories networking: venue: International Agency on Research on Cancer (IARC), Lyon, France, 13–15 December 2017: meeting report. https://www.who.int/publications/i/item/WHO-WHE-CPI-2018.40

[CR145] World Health Organization. 2020. *Laboratory Biosafety Manual*, 4th ed. https://www.who.int/publications/i/item/9789240011311 24404640

[CR146] World Health Organization. 2024. WHO Coronavirus Dashboard. https://covid19.who.int/

[CR147] Wurtz, N., A. Papa, M. Hukic, A. Di Caro, I. Leparc-Goffart, E. Leroy, M.P. Landini, Z. Sekeyova, J.S. Dumler, D. Bădescu, N. Busquets, A. Calistri, C. Parolin, G. Palù, I. Christova, M. Maurin, B. La Scola, and D. Raoult. 2016. Survey of laboratory-acquired infections around the world in biosafety level 3 and 4 laboratories. *European Journal of Clinical Microbiology and Infectious Diseases* 35 (8): 1247–1258. 27234593 10.1007/s10096-016-2657-1 PMC7088173

[CR148] Yeh, K.B., C. Monagin, and J. Fletcher. 2017. Promoting scientific transparency to facilitate the safe and open international exchange of biological materials and electronic data. *Tropical Medicine and Infectious Diseases* 2 (4): 57. 10.3390/tropicalmed2040057 PMC6082060 30270914

[CR149] Young, A. 2023. *Pandora’s Gamble: Lab Leaks, Pandemics, and the World at Risk*. New York: Center Street.

[CR150] Yuan, D., W. Gao, S. Liang, S. Yang, and P. Jia. 2020. Biosafety threats of the rapidly established labs for SARS-CoV-2 tests in China. *Environment International* 143: 105964. 32768807 10.1016/j.envint.2020.105964 PMC7359782

[CR151] Zhou, D., H. Song, J. Wang, Z. Li, S. Xu, X. Ji, X. Hou, and J. Xu. 2019. Biosafety and biosecurity. *Journal of Biosafety and Biosecurity* 1 (1): 15–18. 32501430 10.1016/j.jobb.2019.01.001 PMC7148603

